# Supplementary material for: Validating and prioritizing prenatal breastfeeding education recommendations: A nominal group technique study with postnatal mothers and healthcare professionals
Source: PLoS One. 2025 Jul 16;20(7):e0328542. doi: 10.1371/journal.pone.0328542 (PMC12266410; doi:10.1371/journal.pone.0328542)
Supplement: S1 File — (DOCX) [file pone.0328542.s002.docx]

**S1 File: NGT Discussion Guides**

**Healthcare Practitioners (Lactation Consultant/midwives)**

| **No** | **Questions** | **Illustrative Quotes** |
| --- | --- | --- |
| **1** | The qualitative interview data revealed that although mothers found prenatal breastfeeding education classes beneficial, they still felt unprepared for the actual challenges of breastfeeding. Healthcare practitioners recognize this gap but interpret it in light of their primary objective, which is to encourage mothers to breastfeed. Some of the suggestions from the mothers on how healthcare professionals can better reconcile idealized expectations with their experience of breastfeeding include;  • Presenting a more balanced approach to breastfeeding education to reflect both the advantages and complexities of breastfeeding.  • In-depth discussion regarding the mental and emotional obstacles of breastfeeding, such as “postpartum depression”.  • In-depth discussions addressing the "frustrations and doubts" inherent in the breastfeeding journey, particularly when they are not aligned with planned expectations.  What are your opinions on the mothers' feedback? How do you view this gap, and how practical is it to integrate the mothers' suggestions into future educational sessions? | *“It’s not like we don’t discuss these challenges at all. We mention them, we may not discuss them in depth but we mention them in the class. But we don’t just focus on it. It’s never a priority because, the goal of the classes is to motivate mothers to choose to breastfeed” (LC01).*  *“To be honest, that's a fair critique. We've been so wrapped up in the practical side of things we've maybe lost sight of the emotional journey of breastfeeding. It’s clear we’ve got a bit to learn about balancing the physical and emotional sides of breastfeeding support” (LC03).* |
| **2a**  **2b** | Qualitative interview data highlighted that mother sought more 'interactive' and 'personalized' prenatal breastfeeding education. They recommended using engagement strategies that are 'sensitive' and 'responsive' to their needs. Healthcare practitioners acknowledged this gap, especially considering the pandemic's impact on breastfeeding support. Some of the suggestions from the mothers included;  •Using a pre-class survey to create personalized learning paths.  •Individualized breastfeeding support through one-on-one sessions or smaller group discussions.  •Formalized feedback mechanisms to improve the course content delivery and enhance the learning experience.  •Group educational sessions that Provide opportunities for shared experiences and real-life stories beyond "textbook" information.  Based on the mothers' feedback, how do you perceive this gap, and how practical do you think implementing the mothers' suggestions in future virtual educational sessions?  Data from the qualitative interview indicate that, given the virtual format of the class, mothers highlighted the innovative potential of 'leveraging technology' to provide information and create a more personalized, engaging, and supportive learning environment. Some of their suggestions included using virtual reality scenarios to simulate hands-on breastfeeding techniques, breakout rooms for discussions, and real-time dialogue through indirect communication channels like chat boxes. Healthcare practitioners acknowledged this potential and suggested adopting a 'hybrid model' combining online education with face-to-face sessions. What are your thoughts on these suggestions? How do you perceive the feasibility and effectiveness of incorporating these technological innovations and a hybrid model into future prenatal breastfeeding education sessions? | *- “That’s a fair point. Our current setup, mainly due to the constraints brought on by COVID-19, hasn’t allowed for as much personalized interaction as we’d like. The importance of direct support can’t be understated, especially when it comes to breastfeeding. We're aware this is an area we need to improve on, offering more intimate support structures within the limits of what’s currently safe and feasible” (GC01).*  - *“Yeah. Yeah, I'd say. In all honesty, currently, we do not have a formal feedback mechanism in place for the online classes. This is an area where we could improve” (GC01).*  *-* *It’s a continuous process. We are constantly looking for ways and trying to enhance our online delivery methods to make the virtual experience as enriched as possible. Again, it’s important for me to also say that there are certain aspects we cannot fully replicate in a virtual environment. For that reason, we also provide resources for additional support, like helplines and one-on-one virtual consultations, to bridge the gap as much as we can” (LC03).* |
| **3** | Data from the qualitative interviews indicated that mothers were frustrated by the inconsistent advice during prenatal breastfeeding education classes.  Healthcare practitioners acknowledged that the differences in training and experience among providers contribute to this inconsistency and emphasized the need for continuous professional development. What professional training strategies would you prioritize to address and minimize this inconsistency? | *- “When all staff are on the same page, mothers receive uniform advice regardless of who they speak to. This consistency helps reduce confusion and builds trust in the support system we provide. It's crucial for mothers to feel that everyone involved in their care is working together to support their breastfeeding journey” (CC02* |
| **4** | Data from the qualitative interviews revealed that mothers considered the lack of emphasis on partner attendance a missed opportunity to provide essential breastfeeding support. Healthcare professionals also acknowledged breastfeeding as a 'team effort' and stressed the significance of involving partners 'as much as possible.  What are your thoughts on this feedback from mothers? How practical is integrating this aspect into prenatal breastfeeding education sessions, whether conducted virtually or in person? | **-** *“We're hoping' that by getting' partners involved early on, it'll build a stronger support network for the mammy at home. This should help mammies feel more confident in their decision to start and continue breastfeeding. Knowing they've got the full support of their partner can really take down some of the barriers to breastfeeding” (GC02).* |
| **5** | Data from the qualitative interviews indicated that the mothers highlighted a lack of specific discussion on 'breastfeeding in public' during the classes. Healthcare practitioners acknowledged the importance of addressing this topic, particularly within the 'Irish context.' Given these findings, how practical is incorporating this topic into future prenatal breastfeeding educational sessions? | *“I definitely think it's important to discuss breastfeeding in public in these classes, especially in the Irish context. Ireland has a relatively high breastfeeding initiation rate, but a low rate of breastfeeding continuation, which suggests that many mothers may be facing challenges with breastfeeding in public. By discussing breastfeeding in public and addressing any concerns or fears that mothers may have, we can help to empower them to breastfeed confidently and comfortably in any setting” (RC02).* |

**POSTNATAL MOTHERS**

| **No** | **Questions** | **Illustrative Quotes** |
| --- | --- | --- |
| **1** | Data from the qualitative interviews suggests that the participants found the prenatal breastfeeding education classes helpful. However, they still felt unprepared for the realities of breastfeeding. Some of the suggestions from the participants on how the healthcare professionals can better reconcile idealized expectations with their experience of breastfeeding include;   - Presenting a more balanced approach to breastfeeding education to reflect both the advantages and complexities of breastfeeding. - In-depth discussion regarding the mental and emotional obstacles of breastfeeding, such as “postpartum depression”. - In-depth discussions addressing the "frustrations and doubts" inherent in the breastfeeding journey, particularly when they are not aligned with planned expectations.   Among these suggestions, what strategies do you think could have better prepared you for the experience of breastfeeding? | *-* *It's clear they were trying to promote breastfeeding, and they provided good resources and support for it. But I think they need to give equal attention to the challenges. Make sure mums-to-be are fully prepared for the journey ahead, you know?" (ML01).*  *- “Ah, yes, there were. The advice on how to breastfeed was grand, but I felt they left us a bit at sea about the actual challenges of breastfeeding. Straight after I got home, I was struggling with not enough milk and painful nipples, and I felt completely unprepared for it. It's like those issues were barely touched on in the class, leaving me feeling quite let down, to be honest” (MC01).*  - *“Well, breastfeeding can be very emotional, can't it? With me first, I felt a lot of pressure to get it right, and when it didn’t work out, I felt like I was failing' as a mother. I was hoping' the class would address those feelings, you know, reassure mothers that it’s okay to feel overwhelmed or upset, and give strategies for dealing' with those emotions. But the class was more focused on all the good stuff and all the easy stuff, which is grand, but I was looking for a bit more”* *(MC05).* |
| **2a**  **2b** | From the qualitative interviews, it was clear that participants wanted prenatal breastfeeding education to be more 'interactive' and 'personalized.'. The participants suggested engagement strategies that were 'sensitive' and 'responsive' to their needs, which included:   - Using a pre-class survey to create personalized learning paths. - Individualized breastfeeding support through one-on-one sessions or smaller group discussions. - Formalized feedback mechanisms to improve the course content delivery and enhance the learning experience. - Group educational sessions that Provide opportunities for shared experiences and real-life stories beyond "textbook" information.   Our systematic review revealed that most breastfeeding education interventions in studies reporting optimal breastfeeding outcomes were conducted in groups or clusters of participants, demonstrating the potential effectiveness of group prenatal breastfeeding education in increasing breastfeeding uptake. What interactive strategy would you prioritize among the above suggestions to make the classes more engaging?  Given the virtual format of the classes, the participants highlighted the innovative potential of 'leveraging technology' to provide information and create a more personalized, engaging, and supportive learning environment. Some of the suggestions included;   - Using virtual reality scenarios to simulate hands-on breastfeeding techniques. - Breakout rooms for discussions and real-time dialogue through indirect communication channels like chat boxes.   Findings in our systematic review suggest that computer-based breastfeeding education may be an efficient mode of disseminating breastfeeding information. However, web-based breastfeeding education should be interactive enough to encourage mothers to use it effectively. Considering these suggestions, what digital tools or platforms could enhance the learning experience? | **-** *“You see, breastfeeding can be a very personal and sometimes sensitive topic. Some of us might have had issues or concerns we were a bit embarrassed to voice out loud, especially in a group setting. Having a way to share those thoughts anonymously could really help the educators understand where they might need to adjust the class to better meet our needs. It's about feeling safe to share your true thoughts, you know?” (M R03).*  **-** *“Yeah, there is one more thing I'd like to mention. The online class was convenient for me, I can see how it might not be the best option for everyone. Some expectant mothers might prefer face-to-face classes to have more personalized interactions and immediate answers to their questions. So, having a variety of options available, like both virtual and in-person classes, would be beneficial to cater to different preferences and needs”*  *- “That's one area they could certainly improve. It would have been nice to have an anonymous survey or something of the sort, so that we could have shared our thoughts on the class without feeling embarrassed or singled out” (MG02).*  *- “Well, I'd say make it more interactive, like we've been saying. Use more real-life stories from mums, and maybe even have other moms come in to talk about their experiences. And definitely, more opportunities for us to chat amongst ourselves and with the experts. Make it feel like a community, not just a class. That'd make a world of difference If you ask me” (MC05).*  **-***"I'm reckoning' they could do with more of allowing the mothers to ask questions, I don’t like typing in my questions in the chat box, you know? Maybe get ourselves into smaller group chats where we can have the craic more openly and nab some real-time feedback. And what about a few personalized advice sessions, eh?” (MC01).*  *- “I’d say, try to make the online classes more interactive. Use technology to your advantage, like polls, like question and answer, smaller rooms, I believe you can do that now. I mean create smaller groups within the class you know, stuff like that. And maybe check in with the mums attending, see if they have questions or need to discuss anything. Just because it’s virtual doesn’t mean it can’t be personal and engaging” (MR01).* |
| **3** | Data from the qualitative interviews highlighted that the participants were frustrated by inconsistent advice during prenatal breastfeeding education classes. This inconsistency led to confusion, logistical challenges, and significant emotional stress, resulting in self-doubt and diminished self-confidence. Considering these findings, what are your thoughts on ensuring more consistent and accurate information delivery? Your input will aid in understanding your priorities and improving future educational sessions. | *- “One day, someone would say one thing about how often to feed, and then the next, someone else would say something a bit different. It was a bit confusing, like. It made me feel a bit unsure about the whole thing. I started to wonder if it was going to be more complicated than it was worth” (MR02).* |
| **4** | Data from the qualitative interviews highlighted that many participants feel partner involvement in breastfeeding classes is extremely important. They noted that the current lack of emphasis on partner attendance represents a missed opportunity to provide crucial support for breastfeeding. How much of a priority is partner involvement to you?  Results in our systematic review indicate that family members who are well informed about breastfeeding are more likely to support lactating mothers, increasing their confidence to breastfeed.  How significant do you believe partner participation is in enhancing the effectiveness of these classes? | *- “You see, breastfeeding isn't a solitary task, it’s a family affair, it impacts everyone in the house. The support from the partner, and the family at large, can be critical, especially in the early days” (MC05).* |
| **5** | Data from our qualitative interviews indicated that participants highlighted a lack of specific discussion on 'breastfeeding in public' during the classes. They suggested the need for more practical advice beyond the reassurance that 'it's legal”. Considering the peculiarity of the 'Irish context,' what are your thoughts on this feedback? Is this topic still a priority for you? Would you like this topic to be included in future prenatal breastfeeding education sessions? | *“No, the classes didn't specifically address breastfeeding in public, but I do think it would be helpful for future classes to include information on this topic. I think there's definitely been a shift towards more acceptance of breastfeeding in public in recent years in this country. I also think it's very important for people to be more open to and understanding of breastfeeding in public” (MR02).* |
