# Supplementary material for: Validating and prioritizing prenatal breastfeeding education recommendations: A nominal group technique study with postnatal mothers and healthcare professionals
Source: PLoS One. 2025 Jul 16;20(7):e0328542. doi: 10.1371/journal.pone.0328542 (PMC12266410; doi:10.1371/journal.pone.0328542)
Supplement: S2 Table — (DOCX) [file pone.0328542.s003.docx]

| **GRIPP2 CHECKLIST** | **Item** | **Reported on page No** |
| --- | --- | --- |
| **Section and Topic** |  |  |
| 1. **Aim**   Report the aim of the study. | This study aimed to strengthen the design and relevance of breastfeeding education by incorporating the insights of PPI advisors, including a deputy director of midwifery, a midwife, and a lactation consultant, as active partners. Their expertise informed the development and prioritization of recommendations aligned with the experiences of postnatal mothers. | Yes. Page 3 |
| 1. **Methods**   Provide a clear description of the methods used for PPI in the study. | The PPI group provided vital feedback on the NGT discussion guide, ensuring that the topics and questions directly reflected the priorities identified in previous qualitative study with postnatal mothers and healthcare professionals. They reviewed and refined language, suggested culturally responsive adjustments, and made practical recommendations for recruitment to enhance accessibility and appeal to participants. | Yes. Page 3 |
| 1. **Study results**   Outcomes—Report the results of PPI in the study, including both positive and negative outcomes | The PPI group’s feedback enabled refined, targeted recommendations that accurately addressed postnatal mothers' concerns. Their insights helped adjust the interview guide to be culturally sensitive, practical, and aligned with breastfeeding education. This approach led to data collection that was both meaningful and comfortable for participants. Positive outcomes included enhanced relevance of study findings, though limited time for feedback posed some constraints. | Yes. Page 3 |
| 1. **Discussion and conclusions**   Outcomes—Comment on the extent to which PPI influenced the study overall. Describe the positive and negative effects. | The active involvement of PPI shaped the study in meaningful ways, particularly in creating recommendations that resonated with the real needs of mothers and healthcare practitioners. The partnership enabled us to identify specific areas where mothers’ recommendations aligned with current guidelines, enhancing the study's practical impact. Time limitations for feedback remained a challenge, and future studies might consider extending engagement timelines to support more iterative refinement. | Yes. Page 3 |
| 1. **Reflections/critical perspective**   Critical perspective—Comment critically on the study, reflecting on the things that went well and those that did not, so others can learn from this experience | Reflecting on the process, PPI involvement significantly strengthened the relevance and authenticity of findings. Having advisors from midwifery and lactation provided a balanced, grounded perspective that enriched the recommendations. However, time constraints limited the feedback period, and adapting schedules for better alignment could enhance engagement. The study illustrates that early and flexible PPI involvement deepens research quality and contextual insight. | Yes. Page 3 |

**S2 Table: GRIPP2 short form *(PPI=patient and public involvement)***
