# Supplementary material for: Validating and prioritizing prenatal breastfeeding education recommendations: A nominal group technique study with postnatal mothers and healthcare professionals
Source: PLoS One. 2025 Jul 16;20(7):e0328542. doi: 10.1371/journal.pone.0328542 (PMC12266410; doi:10.1371/journal.pone.0328542)
Supplement: S2 File — (DOCX) [file pone.0328542.s004.docx]

S2 File: Postnatal Mother's Voting card

Prioritizing and Validating Breastfeeding Education Recommendations.

**Instructions for Participants:**

For each question, please review the listed recommendations. Indicate your ranking for each recommendation

Using the following scale:

[ ] Highest Priority (5) [ ] High Priority (4) [ ] Moderate Priority (3) [ ] Low Priority (2) [ ] Lowest Priority (1).

Record your Participant Code below.

***Participant Code****:*

# QUESTION 1

**What do you think would have best prepared you for breastfeeding, considering the need for a balanced approach that addresses both the benefits and challenges, including the mental and emotional aspects like postpartum depression and the frustrations when things don't go as planned?**

- Presenting a more balanced approach to breastfeeding education to reflect both the advantages and complexities of breastfeeding and In-depth discussion regarding the mental and emotional obstacles of breastfeeding, such as “postpartum depression”. [ ]
- Create Informational Resources on Challenges. [ ]
- In-depth discussions addressing the "frustrations and doubts" inherent in the breastfeeding journey, particularly

when they are not aligned with planned expectations. [ ]

# QUESTION 2

**Given your suggestions - such as using a pre-class survey, offering one-on-one support, having a feedback system, and including real-life stories - which interactive strategy would you prioritize to make the classes more engaging, considering the benefits of group-based education?**

- Individualized breastfeeding support through one-on-one sessions or smaller group discussions. [ ]
- Formalized feedback mechanisms to improve the course content delivery and enhance the learning experience. [ ]
- Group educational sessions that Provide opportunities for shared experiences and real-life stories beyond "textbook" information. [ ]
- Using a pre-class survey to create personalized learning paths. [ ]

# QUESTION 3

**What digital tools or platforms could enhance the learning experience, considering suggestions like virtual reality for hands-on techniques, discussion breakout rooms, and chat boxes for real-time dialogue?**

- Using virtual reality scenarios to simulate hands-on breastfeeding techniques. [ ]
- Breakout rooms for discussions and real-time dialogue through indirect communication channels like chat boxes. [ ]

# QUESTION 4

**How can we ensure more consistent and accurate information delivery in prenatal breastfeeding education, given that participants experienced frustration, confusion, and stress due to inconsistent advice?**

- Ongoing professional development for staff to ensure consistent knowledge and adherence to the latest evidence-based information. [ ]
- A standardized handbook or guide for all staff to reference, promoting uniformity in the dissemination of information. [ ]
- Establish standardized guidelines for all midwives and lactation consultants and clear communication practices to ensure consistent advice and build patient confidence without conflicting messages. [ ]
- Routine meetings or consultations among staff to maintain alignment with established guidelines and recent updates. [ ]

# QUESTION 5

**How important do you think partner participation is in improving the effectiveness of breastfeeding classes?**

- Encourage mandatory partner attendance. [ ]
- Offer partner-focused sessions and offer incentives for participation. [ ]
- Integrate family-centered education and provide flexible scheduling. [ ]
- Develop online resources for partners. [ ]

**QUESTION 6**

**Would you consider including practical advice on 'breastfeeding in public,' specifically within the Irish context, as a priority for future prenatal breastfeeding education sessions?**

- Organize public breastfeeding demonstrations. [ ]
- Emphasize legal rights and advocacy. [ ]
- Address cultural and social norms and incorporate practical strategies. [ ]
