# Supplementary material for: Validating and prioritizing prenatal breastfeeding education recommendations: A nominal group technique study with postnatal mothers and healthcare professionals
Source: PLoS One. 2025 Jul 16;20(7):e0328542. doi: 10.1371/journal.pone.0328542 (PMC12266410; doi:10.1371/journal.pone.0328542)
Supplement: S3 Table — (DOCX) [file pone.0328542.s005.docx]

**S3 Table: Postnatal Mothers Priorities Ranking**

| **1.** Presenting a more balanced approach to breastfeeding education to reflect both the advantages and complexities of breastfeeding and In-depth discussion regarding the mental and emotional obstacles of breastfeeding, such as “postpartum depression”. | Highest priority  **5** | High priority  **4** | Highest priority  **5** | Highest priority  **5** | Highest priority  **5** | High priority  **4** | **28** | **PRIORITY** |
| --- | --- | --- | --- | --- | --- | --- | --- | --- |
| **2.** Create Informational Resources on Challenges | High priority  **4** | Moderate priority  **3** | Moderate priority  **3** | Moderate priority  **3** | Moderate priority  **3** | Low priority  **2** | **18** |  |
| **3.** In-depth discussions addressing the "frustrations and doubts" inherent in the breastfeeding journey, particularly when they are not aligned with planned expectations. | Moderate priority  **3** | Highest priority  **5** | High priority  **4** | High priority  **4** | High priority  **4** | Moderate priority  **3** | **23** |  |

| ***QUESTION 2:*** ***Given your suggestions—such as using a pre-class survey, offering one-on-one support, implementing a feedback system, and incorporating real-life stories—which interactive strategy would you prioritize to make the classes more engaging?*** |
| --- |
| **Recommendations**  **Participant 1 Participant 2 Participant 3 Participant 4 Participant 5 Participant 6 Total Score Priority Ranking** |

| **POSTNATAL MOTHERS (N=6)** |
| --- |
| ***QUESTION 1: What do you think would have best prepared you for breastfeeding, considering the need for a balanced approach that addresses both the benefits and challenges, including the mental and emotional aspects like postpartum depression and the frustrations when things don't go as planned?*** |
| **RECOMMENDATIONS AND RANKINGS BASED ON PRIORITIES 25–30 (High Priority)** |
| **Recommendations Participant 1 Participant 2 Participant 3 Participant 4 Participant 5 Participant 6 Total Score Priority Ranking** |

| **1.** Individualized breastfeeding support through one-on-one sessions or smaller group discussions. | Low priority  **2** | Moderate priority  **3** | Moderate priority  **3** | Low priority  **2** | Highest priority  **5** | Low priority  **2** | **17** |  |
| --- | --- | --- | --- | --- | --- | --- | --- | --- |
| **2.** Formalized feedback mechanisms to enhance the delivery of course content and improve the learning experience. | Lowest priority  1 | Low priority  **2** | Low priority  **2** | Lowest priority  **1** | Low priority  **2** | Moderate Priority  **3** | **11** |  |
| **3.** Group educational sessions that provide opportunities for shared experiences and real-life stories beyond "textbook" information. | High priority  **4** | Highest priority  **5** | Highest priority  **5** | Highest priority  **5** | Moderate priority  **3** | Highest priority  **5** | **27** | **PRIORITY** |
| **4.** Using a pre-class survey to create personalized learning paths. | Moderate priority  **4** | High priority  **5** | High priority  **4** | High priority  **5** | High priority  **4** | High priority  **5** | **27** | **PRIORITY** |

| ***QUESTION 3:*** ***What digital tools or platforms could enhance the learning experience, considering suggestions like virtual reality for hands-on techniques, discussion breakout rooms, and chat boxes for real-time dialogue?*** |
| --- |
| **Recommendations Participant 1 Participant 2 Participant 3 Participant 4 Participant 5 Participant 6 Total Score Priority Ranking** |

| **1.** Using virtual reality scenarios to simulate hands-on breastfeeding techniques. | Moderate priority  **3** | High priority  **2** | High priority  **2** | High priority  **4** | High priority  **2** | High priority  **4** | **17** |  |
| --- | --- | --- | --- | --- | --- | --- | --- | --- |
| **2.** Breakout rooms for discussions and real-time dialogue through indirect communication channels, such as chat boxes. | High priority  **4** | Highest priority  **5** | Highest priority  **5** | Highest priority  **5** | Highest priority  **5** | Highest priority  **5** | **29** | **PRIORITY** |

| ***QUESTION 4******: How can we ensure more consistent and accurate information delivery in prenatal breastfeeding education, given that participants experienced frustration, confusion, and stress due to inconsistent advice?*** |
| --- |
| **Recommendations Participant 1 Participant 2 Participant 3 Participant 4 Participant 5 Participant 6 Total Score Priority Ranking** |

| **1.** Ongoing professional development for staff to ensure consistency in knowledge and adherence to the latest evidence-based information. | High priority  **4** | High priority  **4** | High priority  **4** | High priority  **4** | High priority  **4** | High priority  **4** | **24** |  |
| --- | --- | --- | --- | --- | --- | --- | --- | --- |
| **2.** A standardized handbook or guide for all staff to reference, promoting uniformity in the dissemination of information. | Moderate priority  **3** | Low priority  **2** | Low priority  **2** | Moderate priority  **3** | Low priority  **2** | Moderate priority  **3** | **15** |  |
| **3.** Establish standardized guidelines for all midwives and lactation consultants and clear communication practices to ensure consistent advice and build patient confidence without conflicting messages | Highest priority  **5** | Highest priority  **5** | Highest priority  **5** | Highest priority  **5** | Highest priority  **5** | Highest priority  **5** | **30** | **PRIORITY** |
| **4.** Routine meetings or consultations among staff to maintain alignment with established guidelines and recent updates. | Low priority  **2** | Moderate priority  **3** | Moderate priority  **3** | Low priority  **2** | Moderate priority  **3** | Lowest priority  **1** | **14** |  |

| ***QUESTION 5:*** ***How important do you think partner participation is in improving the effectiveness of breastfeeding classes?*** |
| --- |
| **Recommendations Participant 1 Participant 2 Participant 3 Participant 4 Participant 5 Participant 6 Total Score Priority Ranking** |

| **1.** Encourage mandatory partner attendance | Low priority  **2** | Low priority  **2** | Lowest priority  **1** | Low priority  **2** | Low priority  **2** | Lowest priority  **1** | **10** |  |
| --- | --- | --- | --- | --- | --- | --- | --- | --- |
| **2.** Offer partner-focused sessions and offer incentives for participation | Moderate priority  **3** | High priority  **4** | Moderate priority  **3** | High priority  **4** | High priority  **4** | Moderate priority  **4** | **22** |  |
| **3.** Integrate family-centred education (Partner Involvement) and provide flexible scheduling. | Highest priority  **5** | Highest priority  **5** | Highest priority  **5** | Highest priority  **5** | Highest priority  5 | Highest priority  **5** | **30** | **PRIORITY** |
| **4.** Develop online resources for partners | High priority  **4** | Moderate priority  **4** | Low priority  **2** | Moderate priority  **3** | Lowest priority  **1** | Low priority  **2** | **17** |  |

| ***QUESTION 6:*** ***Would you consider including practical advice on "breastfeeding in public," specifically within the Irish context, as a priority for future prenatal breastfeeding education sessions?*** | | | | | | | | |
| --- | --- | --- | --- | --- | --- | --- | --- | --- |
| **Recommendations Participant 1 Participant 2 Participant 3 Participant 4 Participant 5 Participant 6 Total Score Priority Ranking** | | | | | | | | |
| **1.**Organize Public Breastfeeding Demonstrations | Low priority  **2** | Lowest priority  **1** | Low priority  **2** | Low priority  **2** | Low priority  **2** | Lowest priority  **1** | **10** |  |
| Emphasize Legal Rights and Advocacy | Moderate priority  **3** | High priority  **4** | Moderate priority  **3** | Lowest priority  **1** | Moderate priority  **3** | Low priority  **2** | **16** |  |
| **2.**Addressing Breastfeeding in Public: Empowering Mothers Through Practical Support and Open Dialogue | Highest priority  **5** | High priority  **4** | Highest priority  **5** | High priority  **4** | Highest priority  **5** | Moderate priority  **3** | **26** | **PRIORITY** |

***Each postnatal mother ranked the recommendations on a descending scale from 5 (highest priority) to 1 (lowest). Aggregate scores were calculated to identify group priorities, with tied scores resolved by the frequency of votes. Recommendations with cumulative scores between 25–30 were classified as high priority. Of the 20 recommendations presented, seven were prioritised by the postnatal mothers.***
