# Supplementary material for: Validating and prioritizing prenatal breastfeeding education recommendations: A nominal group technique study with postnatal mothers and healthcare professionals
Source: PLoS One. 2025 Jul 16;20(7):e0328542. doi: 10.1371/journal.pone.0328542 (PMC12266410; doi:10.1371/journal.pone.0328542)
Supplement: S3 File — (DOCX) [file pone.0328542.s006.docx]

S3 File: Healthcare Professionals Voting Card

Prioritizing and Validating Breastfeeding Education Recommendations.

**Instructions:** Please fill in the feasibility ranking and indicate the corresponding step(s) of the "10 Steps to Successful Breastfeeding" for each priority.

*Participant code* **-------------------------**

**Feasibility Ranking**: For each priority, please rank how feasible it would be to incorporate it into the current prenatal breastfeeding education program using the following scale:

1. **Not Feasible**
2. **Slightly Feasible**
3. **Moderately Feasible**
4. **Highly Feasible**
5. **Very Highly Feasible**
6. **Alignment with the 10 Steps to Successful Breastfeeding**: Indicate which of the "10 Steps to Successful Breastfeeding" this priority would fall under, if applicable. The "10 Steps" are:
   1. Step 1: Have a written breastfeeding policy that is routinely communicated to all healthcare staff.
   2. Step 2: Train all healthcare staff in the skills necessary to implement this policy.
   3. Step 3: Inform all pregnant women about the benefits and management of breastfeeding.
   4. Step 4: Help mothers initiate breastfeeding within half an hour of birth.
   5. Step 5: Show mothers how to breastfeed and maintain lactation, even if they are separated from their infants.
   6. Step 6: Give newborn infants no food or drink other than breast milk unless medically indicated.
   7. Step 7: Practice rooming-in – allow mothers and infants to remain together 24 hours a day.
   8. Step 8: Encourage breastfeeding on demand.
   9. Step 9: Do not give breastfeeding infants artificial teats or pacifiers (also called dummies or soothers).
   10. Step 10: Establish breastfeeding support groups and refer mothers to them on discharge from the hospital or clinic.

| **Priority** | **Feasibility Ranking** | **Alignment with the 10 Steps** |
| --- | --- | --- |
| 1. Presenting a more balanced approach to breastfeeding education to reflect both the advantages and complexities of breastfeeding and In-depth discussion regarding the mental and emotional obstacles of breastfeeding, such as “postpartum depression. | 1. Not Feasible [ ]  2. Slightly Feasible [ ]  3. Moderately Feasible [ ]  4. Highly Feasible [ ]  5. Very Highly Feasible [ ] | [Step X] |
| 1. Breakout rooms for discussions and real-time dialogue through indirect communication channels like chat boxes. | 1. Not Feasible [ ]  2. Slightly Feasible [ ]  3. Moderately Feasible [ ]  4. Highly Feasible [ ]  5. Very Highly Feasible [ ] | [Step X] |
| 1. Group educational sessions that Provide opportunities for shared experiences and real-life stories beyond "textbook" information. | 1. Not Feasible [ ]  2. Slightly Feasible [ ]  3. Moderately Feasible [ ]  4. Highly Feasible [ ]  5. Very Highly Feasible [ ] | [Step X] |
| 1. Using a pre-class survey to create personalized learning paths. | 1. Not Feasible [ ]  2. Slightly Feasible [ ]  3. Moderately Feasible [ ]  4. Highly Feasible [ ]  5. Very Highly Feasible [ ] | [Step X] |
| 1. Establish standardized guidelines for all midwives and lactation consultants and clear communication practices to ensure consistent advice and build patient confidence without conflicting messages | 1. Not Feasible [ ]  2. Slightly Feasible [ ]  3. Moderately Feasible [ ]  4. Highly Feasible [ ]  5. Very Highly Feasible [ ] | [Step X] |
| 1. Integrate family-centered education and provide flexible scheduling. | 1. Not Feasible [ ]  2. Slightly Feasible [ ]  3. Moderately Feasible [ ]  4. Highly Feasible [ ]  5. Very Highly Feasible [ ] | [Step X] |
| 1. Address cultural and social norms and incorporate practical strategies. | 1. Not Feasible [ ]  2. Slightly Feasible [ ]  3. Moderately Feasible [ ]  4. Highly Feasible [ ]  5. Very Highly Feasible [ ] | [Step X] |
