# Supplementary material for: Validating and prioritizing prenatal breastfeeding education recommendations: A nominal group technique study with postnatal mothers and healthcare professionals
Source: PLoS One. 2025 Jul 16;20(7):e0328542. doi: 10.1371/journal.pone.0328542 (PMC12266410; doi:10.1371/journal.pone.0328542)
Supplement: S4 Table — (DOCX) [file pone.0328542.s007.docx]

| **Healthcare Professionals (Lactation consultants/Midwives) (N=4)** |
| --- |
| **Prioritised Recommendations, Feasibility and Alignment with the WHO's 10 Steps to Successful Breastfeeding.**  **18–20 (Feasible)** |
| **Prioritised**  **Recommendation Participant 1 Participant 2 Participant 3 Participant 4 Total Score Feasibility Ranking Alignment with the 10 Steps To**  **Successful Breastfeeding.** |

**S4 Table: Feasibility of Incorporating Prioritized Recommendations into the Prenatal Breastfeeding Education Guide and Their Alignment with the WHO's 10 Steps to Successful Breastfeeding.**

|  | **Presenting a more balanced approach to breastfeeding education to reflect both the advantages and complexities of breastfeeding, and In-depth discussion regarding the mental and emotional obstacles of breastfeeding, such as “postpartum depression.** | Very Highly Feasible  **5** | Highly Feasible  **4** | Very Highly Feasible  **5** | Very Highly Feasible  **5** | **19** | **FEASIBLE** | **Steps 3 and step 5 of the WHO's 10 Steps to Successful Breastfeeding.** |
| --- | --- | --- | --- | --- | --- | --- | --- | --- |
|  | **Breakout rooms for discussions and real-time dialogue through indirect communication channels like chat boxes.** | Moderately Feasible  **3** | Very Highly Feasible  **5** | Moderately Feasible  **3** | Very Highly Feasible  **5** | **15** | **NOT FEASIBLE** | **Step 3 of the WHO's 10 Steps to Successful Breastfeeding** |
|  | **Group educational sessions that offer opportunities for shared experiences and real-life stories, complementing textbook information.** | Very Highly Feasible  **5** | Very Highly Feasible  **5** | Very Highly Feasible  **5** | Very Highly Feasible  **5** | **20** | **FEASIBLE** | **Step 3 of the WHO's 10 Steps to Successful Breastfeeding.** |
|  | **Using a pre-class survey to create personalized learning paths.** | Very Highly Feasible  **5** | Very Highly Feasible  **5** | Very Highly Feasible  **5** | Very Highly Feasible  **5** | **20** | **FEASIBLE** | **Steps 2 and 3 of the WHO's 10 Steps to Successful Breastfeeding.** |
|  | **Establish standardized guidelines for all midwives and lactation consultants and clear communication practices to ensure consistent advice and foster patient confidence by avoiding conflicting messages.** | Very Highly Feasible  **5** | Very Highly Feasible  **5** | Very Highly Feasible  **5** | Very Highly Feasible  **5** | **20** | **FEASIBLE** | **Step 2 of the WHO's 10 Steps to Successful Breastfeeding.** |
|  | **Integrate family-centred education (Partner Involvement) and provide flexible scheduling.** | Moderately Feasible  **3** | Moderately Feasible  **3** | Moderately Feasible  **3** | Moderately Feasible  **3** | **12** | **NOT FEASIBLE** | **Healthcare professionals acknowledged the value of partner involvement but found real-time integration challenging. They recommended incorporating flexible, asynchronous resources into Step 3 to support partner engagement, thereby laying the groundwork for involvement in later stages such as Steps 5 and 7.** |
| **7)** | **Addressing Breastfeeding in Public: Empowering Mothers Through Practical Support and Open Dialogue** | Very Highly Feasible  **5** | Highly Feasible  **4** | Very Highly  Feasible  **5** | Very Highly Feasible  **5** | **19** | **FEASIBLE** | **This recommendation was deemed to be feasibly implementable and can be incorporated into the current prenatal breastfeeding education framework in Ireland by organically discussing it in the classes. However, the healthcare professionals determined that this recommendation does not align seamlessly with any of the World Health Organisation's (WHO) 10 steps to successful breastfeeding.** |
