# Supplementary material for: Validating and prioritizing prenatal breastfeeding education recommendations: A nominal group technique study with postnatal mothers and healthcare professionals
Source: PLoS One. 2025 Jul 16;20(7):e0328542. doi: 10.1371/journal.pone.0328542 (PMC12266410; doi:10.1371/journal.pone.0328542)
Supplement: S5 Tables — (DOCX) [file pone.0328542.s008.docx]

**S5 Tables on Framework Matrix Based on Ritchie Spencers Framework Analysis**

**Table (5a):** **Framework Matrix Based on Ritchie & Spencer’s Framework Analysis**

| **Theme** | **Familiarization (Quotes)** | **Identifying a Thematic Framework** | **Indexing (Code Examples)** | **Charting (Summary by Participant)** | **Mapping & Interpretation (WHO Alignment)** |
| --- | --- | --- | --- | --- | --- |
| Balanced Breastfeeding Education, including  Mental & Emotional Preparedness | M1: “If I had known more about the ups and downs, I would not have been so shocked when things got tough.” LCC1: “Adding a bit about the challenges would not be too hard, right?”  M3: “I was not expecting to feel so overwhelmed…”  LCC4: “If we’re looking at what’s easiest to implement first…” | Need for balanced, realistic education in prenatal sessions.  Normalizing doubt and emotional challenges in breastfeeding. | Benefits vs. Challenges, Misalignment of Expectations, Realistic Preparation.  Overwhelm, Emotional Support, Mental Load, Postnatal Confidence | All Mothers (M1-M6) requested less idealized info; LCCs agreed with integrating into the current Step 3.  All mothers supported immediate inclusion (M1-M6) while LCCs preferred gradual integration post-partum. | WHO Step 3 – Educate on breastfeeding benefits and management.  WHO Step 5 – Support mothers in initiating and continuing breastfeeding. |
| Real-Time Dialogue via Breakout Rooms and Indirect Communication Channels (e.g., Chat Functions) | “Sometimes you just need to ask something without stopping the whole class—and it’s great to scroll back and see what others asked too” (M4).  “It’s a great idea in theory, but it’s challenging to teach and manage a busy chat simultaneously without missing something important” (LCC2) | Supportive space for participants to engage, ask questions, or respond to information provided in the classes. | Practical enhancements that facilitated questions and clarifications without disrupting the flow of the session. | All mothers (M1-M6) emphasized the value of real-time, indirect communication tools, such as chat functions, within breastfeeding education sessions.  All LCCs recognized the value of chat functions in encouraging engagement, especially among quieter participants. However, they rated it lower in feasibility due to concerns that managing both verbal and written communication in real time could hinder effective content delivery. | WHO Step 3-Discuss the importance and management of breastfeeding with pregnant women and their families |
| Group educational sessions that provide opportunities for shared experiences | “When you hear other mums talk honestly about the tough bits and the little wins—it just makes you feel like you’re not alone in it” (M5). | Group Educational Sessions Featuring Real-Life Stories and Shared Experiences | Effective in alleviating feelings of isolation, strengthening peer relationships, and offering practical insights beyond standard instructional material. | All mothers (M1-M6) described these narratives as both emotionally affirming and practically informative, contributing to the normalization of the breastfeeding experience. Healthcare professionals similarly acknowledged the importance of genuine maternal stories in fostering a supportive and relatable educational setting. | WHO Step 3-Discuss the importance and management of breastfeeding with pregnant women and their families |
| Personalized Learning via Pre-Class Surveys | M6: “It’s all about making’ sure the information we’re getting’ is actually relevant...” LCC1: “We could tailor the content a bit more…” | Use of surveys to guide class structure and tailor messaging | Tailored Support, Survey Feedback, Relevance of Content | Mothers valued customization. LCCs suggested pairing the survey with staff training. | WHO Steps 2  – Train staff and deliver tailored education.  WHO Step 3-Discuss the importance and management of breastfeeding with pregnant women and their families |
| Consistency in Breastfeeding Advice | M6: “It’d help if they had a standard set of guidelines...” LCC3: “A handbook is a great reference, but training matters too.” | Standardized messaging across professionals | Conflicting Information, Standardized Guidance, Unified Training | Mothers stressed handbook; LCCs leaned towards staff mentorship & training. | WHO Step 2 – Train healthcare staff effectively |
| Partner Involvement | M1: “If the partner is involved… they can offer real support.” LCC2: “Offering recorded sessions or online modules…” | Enhancing the partner’s supportive role through flexible options | Support Roles, Flexibility, Inclusion, Emotional Help | Mothers advocated live support; LCCs proposed asynchronous methods. | Practical constraints, such as partners’ work schedules, limited consistent participation in prenatal education. Although flexible strategies were viewed as inclusive and effective, they fall outside the scope of the WHO’s Ten Steps, which do not explicitly address partner involvement. |
| Breastfeeding in Public | M4: “It’s grand to be told it’s legal, but that doesn’t make it easier…” LCC1: “It’s hard to see where it would fit…” | Addressing stigma, discomfort and public confidence | Stigma, Cultural Norms, Confidence, Role-Playing | Mothers requested scenario-based tools; LCCs preferred open discussions. | Not specific to WHO steps; recommend informal integration into general sessions |

# Table (5b): Revised Framework Matrix Based on Ritchie & Spencer’s Framework Analysis (Stage 4)

| **Theme** | **Framework Stage** | **Participants** | **LCCs Response Summary** | **Mother Response Summary** | **WHO 10 Step Alignment** | **LCCs Quotes** | **Mother Quotes** |
| --- | --- | --- | --- | --- | --- | --- | --- |
| Balanced prenatal breastfeeding education.  Mental and emotional preparedness (normalization of frustrations and doubts) | Charting | LCC1, LCC2, LCC3, LCC4, M1-M6 | LCCs found a balanced approach feasible within Step 3, aiming to set realistic expectations without discouraging mothers.  LCCs acknowledged the importance of emotional support but preferred a phased introduction starting with practical challenges. | Mothers strongly valued balanced preparation covering both benefits and difficulties.  Mothers prioritized emotional and mental health support during breastfeeding | Steps 3 and 5. | “Adding a bit about the challenges would not be too hard, right?” (LCC1).  “We need to normalize those struggles a bit more, so they don’t feel like they’re on their own” (LCC3) | “If I had known more about the ups and downs, I wouldn’t have been so shocked when things got tough” (M1).  “I wasn’t expecting to feel so overwhelmed… I’d have been more prepared” (M3) |
| Personalized learning through pre-class surveys | Charting | LCC1, LCC2, LCC3, LCC4, M1-M6 | LCCs saw the pre-class survey as practical and easily implementable to personalize learning. | Mothers supported tailored content through surveys for relevance. | Steps 2 & 3 | “That way, we could tailor the content a bit more, make it more relevant to them” (LCC1) | “It’s all about making’ sure the information we’re getting’ is actually relevant” (M6) |
| Group educational sessions with real-life stories | Charting | LCC1, LCC2, LCC3, LCC4, M1-M6 | LCCs valued real-life stories but prioritized structured group discussions due to resource constraints. | Mothers appreciated shared stories and preferred open discussion formats. | Step 3 | “If we know what the mothers are going' through, we can bring in more relevant examples” (LCC1) | “When ye hear other mums talking’ about their experiences, it just hits home” (M5) |
| Digital tools: breakout rooms and chat boxes | Charting | LC1, LC2, LC3, LC4, M1-M6 | LCCs noted logistical challenges in breakout rooms and chat boxes in Steps 3. | Mothers highly prioritized real-time support formats like breakout rooms. | Step 3 | “Breakout rooms and chat support would be fantastic, but realistically, it's hard to manage” (LCC4) | “In a big group, it’s easy to feel a bit lost… but in a smaller group, you can ask freely” (M2) |
| Consistent and accurate breastfeeding guidance | Charting | LCC1, LCC2, LCC3, LCC4, M1-M6 | LCCs proposed standardized training, communication strategies, and mentorship under Step 2. | Mothers emphasized the need for consistent information to reduce confusion. | Step 2 | “Standardized training is a must… We need to be sure we’re all explaining’ things in a clear way” (LCC1) | “It’d help if they had a standard set of guidelines all stick to” (M6) |
| Partner involvement in breastfeeding education | Charting | LCC1, LCC2, LCC3, LCC4, M1-M6 | LCCs valued partner involvement but suggested flexible options due to attendance challenges. | Mothers wanted partners actively involved for better emotional support. | The WHO’s 10 Steps do not explicitly address partner involvement. | “Flexible options, like recorded sessions, might work better given the reality of partners’ schedules” (LCC1) | “If the partner is involved, they get to understand what we’re going through and offer real support” (M1) |
| Breastfeeding in public support | Charting | LCC1, LCC2, LCC3, LCC4, M1-M6 | The LCCs agreed it's important but suggested discussing it organically due to its lack of fit in the 10 Steps. | Mothers wanted practical support and normalization of public breastfeeding. | Discussed informally – not directly in a step | “We could bring it up more organically by asking the mothers to share their concerns” (LCC3) | “It’s grand to be told it’s legal, but that doesn’t make it any easier” (M4) |
