# Supplementary material for: Validating and prioritizing prenatal breastfeeding education recommendations: A nominal group technique study with postnatal mothers and healthcare professionals
Source: PLoS One. 2025 Jul 16;20(7):e0328542. doi: 10.1371/journal.pone.0328542 (PMC12266410; doi:10.1371/journal.pone.0328542)
Supplement: S6 Table — (DOCX) [file pone.0328542.s009.docx]

**S6 Table: Summary of Findings**

| **Nos** | **CONSENSUS PRIORITIES FROM POSTNATAL MOTHERS** | **FEASIBILITY OF IMPLEMENTATION BY HEALTHCARE PROFESSIONALS** | **ALIGNMENT WITH KEY STEPS OF THE WHO’S 10 STEPS TO SUCCESSFUL BREASTFEEDING** |
| --- | --- | --- | --- |
| **1** | **Balanced Prenatal Breastfeeding Education with Emphasis on Mental and Emotional Preparedness (Normalizing Frustration and Self-Doubt).** | Healthcare professionals agreed that a more balanced approach is feasible to implement, particularly within the WHO Step 3 framework, which covers the benefits and management of breastfeeding. | Healthcare professionals agreed that this recommendation aligns most closely with Steps 3 and 5 of the WHO’s 10 Steps to Successful Breastfeeding, which focus on informing mothers about the benefits and management of breastfeeding, supporting mothers with breastfeeding initiation, and providing ongoing assistance. |
| **2** | **Prioritizing Personalized Learning Through Pre-Class Surveys.** | Healthcare professionals agreed that a pre-class survey would be a feasible first step toward personalization. They recognized it as a manageable addition that wouldn't require extensive restructuring | Healthcare professionals identified Step 2 (staff training) and Step 3 (education on breastfeeding benefits and management) as suitable points for integrating the pre-class survey. |
| **3** | **Group Educational Sessions Featuring Real-Life Stories and Shared Experiences.** | Healthcare professionals recognized the value of authentic maternal narratives in creating a supportive and relatable learning environment. However, they emphasized that the feasibility of incorporating structured storytelling into group sessions would depend on gaining insight into participants’ specific needs | The healthcare professionals agreed that this recommendation was most closely aligned with Step 3, which focuses on informing mothers about the benefits and management of breastfeeding. |
| **4** | **Real-Time Dialogue via Breakout Rooms and Indirect Communication Channels (e.g., Chat Functions).** | Healthcare professionals acknowledged the potential of chat functions to support real-time engagement, particularly for participants less inclined to speak up in large groups. However, they ranked the priority lower in feasibility due to concerns about managing dual communication streams during live sessions. | The healthcare professionals agreed that this recommendation aligns with Step 3, which emphasizes initiating and maintaining breastfeeding through ongoing support and problem-solving. |
| **5** | **Ensuring Consistent and Accurate Breastfeeding Guidance Through Standardization.** | Healthcare professionals agreed that achieving consistency across providers was feasible through a multifaceted approach that included standardized training, clear communication strategies, mentorship, and shared resources. | This recommendation aligns most directly with Step 2, which focuses on staff training to support breastfeeding. |
| **6** | **Partner Involvement as a Supportive Component in Breastfeeding Education.** | Healthcare professionals ranked partner involvement lower in priority due to concerns about its feasibility within the constraints of the existing class structure. | Healthcare professionals supported partner involvement but found real-time integration impractical. They recommended flexible, asynchronous resources introduced in Step 3 to support partner engagement, gradually preparing them for later stages such as breastfeeding initiation and ongoing support in Steps 5 and 7. |
| **7** | **Addressing Breastfeeding in Public: Empowering Mothers Through Practical Support and Open Dialogue.** | Healthcare professionals acknowledged the cultural sensitivity and relevance of breastfeeding in public. Still, they expressed reservations about incorporating it into the formal structure of prenatal education as defined by the WHO 10 Steps to Successful Breastfeeding. | Unlike the other prioritized recommendations identified in this study, public breastfeeding does not directly align with any of the WHO’s 10 Steps to Successful Breastfeeding. |
