# Supplementary material for: Validating and prioritizing prenatal breastfeeding education recommendations: A nominal group technique study with postnatal mothers and healthcare professionals
Source: PLoS One. 2025 Jul 16;20(7):e0328542. doi: 10.1371/journal.pone.0328542 (PMC12266410; doi:10.1371/journal.pone.0328542)
Supplement: Attachment — Submitted filename: Response to Reviewers.docx [file pone.0328542.s011.docx]

**Manuscript Ref. No: PONE-D-25-09271**

**Responses to reviewers’ comments on Title:** *Validating and Prioritizing Prenatal Breastfeeding Education Recommendations: A Nominal Group Technique Study with Postnatal Mothers and Healthcare Professionals.*

We would like to express our sincere gratitude to the editor and the three reviewers for their time and efforts in providing detailed, insightful, and valuable feedback on our manuscript. Their suggestions have helped to sharpen our thinking and, we hope, led to a substantially improved submission based on their valuable constructive feedback.

| **Editor’s Comments** | **Response** |
| --- | --- |
| If they DID provide consent for these transcripts to be published, please also confirm that the transcripts do not contain any potentially identifying information (or let us know if the participants consented to having their personal details published and made publicly available). We consider the following details to be identifying information:  - Names, nicknames, and initials  - Age more specific than round numbers  - GPS coordinates, physical addresses, IP addresses, and email addresses  - Information in small sample sizes (e.g., 40 students from X class in X year at X university)  - Specific dates (e.g., visit dates, interview dates)  - ID numbers  Or, if the participants DID NOT provide consent for these transcripts to be published:  - Provide a de-identified version of the data or excerpts of interview responses  - Provide information regarding how these transcripts can be accessed by researchers who meet the criteria for access to confidential data, including:  a) the grounds for restriction  b) The name of the ethics committee, Institutional Review Board, or third-party organization that is imposing sharing restrictions on the data  c) A non-author, institutional point of contact that can field data access queries, in the interest of maintaining long-term data accessibility.  d) Any relevant data set names, URLs, DOIs, etc. that an independent researcher would need to request in order to access your minimal data set. | Thank you for your valuable comment, and we appreciate the opportunity to clarify this feedback regarding participant consent and qualitative data handling. We confirm that all participants provided written informed consent before data collection. This consent process included explicit agreement for anonymized narrative quotes to be used in publications and presentations arising from the research and we have clarified this under ***Recruitment, page 11.*** |
| For further information on sharing data that contains sensitive participant information, please see:  1. A complete description of the dataset | Thank you for raising this important point.  While participants provided verbal and written informed consent, including consent for using anonymized quotes in publications, ethical approval for this study did not extend to publishing the full interview transcripts per participant. This approach was explicitly discussed during pre-approval consultations with the Research Ethics Committees of the participating sites, where it was agreed that only selected, de-identified excerpts or participant quotes would be included in any dissemination materials to safeguard participant anonymity and confidentiality while minimizing the risk of deductive disclosure. This is particularly relevant in a healthcare setting involving professionals and service users from identifiable institutions. Therefore, consistent with established ethical guidelines and data protection principles, the manuscript presents only thematically relevant and anonymized quotes, using coded identifiers (e.g., M1 for postnatal mothers, LCC1 for healthcare professionals/Lactation Consultants) to preserve confidentiality while illustrating key findings. |
| 2. The nature of the restrictions upon the data (ethical, legal, or owned by a third party) and the reasoning behind them | The dataset contains sensitive, context-specific information drawn from a small sample, and even with pseudonymization, the risk of deductive disclosure remains. As a result, public access to full transcripts is restricted based on ethical considerations. The anonymized excerpts included in the manuscript are identified only by general codes (e.g., M1 for postnatal mothers, LCC1 for healthcare professionals) to preserve participant confidentiality. |
| 3. The full name of the body imposing the restrictions upon your dataset (ethics committee, institution, data access committee, etc) | Requests for access to the underlying data may be submitted to the overseeing ethics body, subject to institutional review and appropriate safeguards:   - **Grounds for restriction**: Ethical protection of participant confidentiality due to the nature of the data. - **Reviewing ethics committee**: Health Service Executive (HSE) Research Ethics Committees of three tertiary hospital sites in the Republic of Ireland (University Maternity Hospital Limerick- REC Ref: 119/2021, Rotunda Maternity Hospital Dublin-REC-2021-027, University Hospital Galway-Ref: C.A. 2718). Each of their contact details are in the approval letter for each site. |
| 4. If the data are owned by a third party, confirmation of whether the authors received any special privileges in accessing the data that other researchers would not have | Data is not owned by a third party |
| 5. Direct, non-author contact information (preferably email) for the body imposing the restrictions upon the data, to which data access requests can be sent | **Reviewing ethics committee**: Health Service Executive (HSE) Research Ethics Committees of three tertiary hospital sites in the Republic of Ireland (University Maternity Hospital Limerick- REC Ref: 119/2021, Rotunda Maternity Hospital Dublin-REC-2021-027, University Hospital Galway-Ref: C.A. 2718).  **Institutional contact for data access**: Annmarie.Grealish@ul.ie.  We hope this clarifies the ethical rationale for the restricted access to the full dataset. |
| In the ethics statement in the Methods, you have specified that verbal consent was obtained. Please provide additional details regarding how this consent was documented and witnessed, and state whether this was approved by the IRB | Thank you for highlighting this and we appreciate the opportunity to clarify your valuable comment regarding the consent process. We obtained written informed consent from all participants before they participated in both the interviews and the NGT sessions. Additionally, verbal consent was obtained immediately before the NGT sessions as an ethical safeguard and procedural confirmation, particularly given the virtual setting of the group discussions. The verbal consent process was conducted in accordance with institutional and ethical guidelines and approved by the Health Service Executive (HSE) Research Ethics Committees of the three tertiary hospital sites in the Republic of Ireland (Reference Numbers: University Maternity Hospital Limerick- REC Ref: 119/2021, Rotunda Maternity Hospital Dublin-REC-2021-027, University Hospital Galway-Ref: C.A. 2718). The verbal consent served as an additional confirmation of informed participation, not a substitute for written consent. Based on your valuable comment we have now amended and clarified this under ***Recruitment, page 11, and Ethical approval, page 19*** and we have reported that both written and verbal consent were obtained. |
| The objective of the study is to identify and prioritize recommendations for breastfeeding education. The results should therefore be clearer on what recommendations were identified and how they were prioritized. The results showed emergent themes, which, in my opinion, do not address your objectives. | Thank you for your insightful comment. We appreciate your observation that the Results section, as originally presented, could be more clearly aligned with the study’s objective: to validate, refine, and prioritise recommendations for improving prenatal breastfeeding education. For clarity, the transcripts from the Nominal Group Technique (NGT) sessions were examined using a two-phased qualitative analytical framework under ***Data analysis pages 18-19***:   1. *Content Analysis* (Kyngäs, 2019; Graneheim & Lundman, 2004) was initially applied to categorize and summarize the participants' prioritized ideas. This involved grouping similar recommendations and identifying the frequency of mentions across participants ***(See Data analysis, page 18).*** 2. *Ritchie’s Framework Analysis* (Ritchie et al., 2014) was subsequently employed to explore these prioritized items in greater depth which is described under ***Data analysis, see page 19*.** A matrix chart **(See S5 Tables)** was developed to chart responses across the two groups (postnatal mothers and healthcare professionals), mapping how participants explained or justified their choices. This enabled comparison while preserving both context and meaning. This has been further explained in details in ***Data analysis, page 19.***   Using these two methods in tandem allowed us to balance breadth and depth: Content Analysis captured the scope and recurrence of ideas, while Framework Analysis provided a structured interpretation of the rationale behind participant priorities. We acknowledge that using "emergent themes" may have introduced some ambiguity. These themes did not arise inductively from raw qualitative data; they were the product of participant interaction and consensus-building during the refinement and prioritization of recommendations developed in our previous study. We have clarified this in the revised manuscript under ***Results (emergent themes), page 24*** to emphasize that the themes reflect the outcomes of structured, deliberative group processes, rather than traditional thematic analysis. In response to your comment, the Results section has been revised to align more clearly with the study’s objective and based on your comments we revised the summary of findings in ***S6 Table*** which shows the final prioritized recommendations from the postnatal mothers. |
| PLOS does not require blinding of previously conducting research and operates via open peer review. There is no need to redact the previous study and might be better if you cite them here so we can assess anything that needs to be assessed. | Thank you for this clarification and based on your valuable comment that PLOS does not require anonymization of previously conducted or related research due to its open peer review model, we have now removed the blinding and have now explicitly cited the prior qualitative study (Kehinde et al., 2024) that informed the development of the 20 recommendations evaluated in this current NGT study. This is the qualitative paper <https://doi.org/10.1371/journal.pone.0315269>. This citation has been added to the **Introduction** and **Methods** sections ***(see page 7 and pages 9-10*)** and other relevant sections in the manuscript which allows for appropriate contextualization and assessment of methodological continuity where the transition from our previous qualitative study to this NGT study is discussed. |
| Recommendations should not be bulleted and should still be presented narratively. | Thank you for highlighting this point. Based on your valuable comment, we fully amended and revised this section under ***Recommendations, pages 43-52***. We removed the bullet points, and it is now fully written and presented in narrative style, in line with PLOS guidelines. |
| Please shorten your paper as it is too long as it stands. | Thank you for your comment regarding the manuscript length. This paper presents findings from a NGT study, which requires detailed methodological reporting and careful presentation of rich, multi-perspective qualitative data. In line with established qualitative research standards, we have aimed to ensure analytical depth, transparency, and replicability. While PLOS ONE does not impose a word limit, we have maintained clarity and conciseness. We also note that additional explanatory content has been added in response to reviewer feedback, including requests for further clarification in the limitations section and results. As such, we respectfully maintain that the current length is appropriate for the study's complexity and significance. |
| Add a limitations section to your manuscript. | Thank you for highlighting this point and based on your valuable comment we have now included ***Limitations*** *(****see page 52)*** section in the revised manuscript.  Many thanks for you feedback and we would like to express our sincere gratitude for your time and efforts in providing detailed, insightful, and valuable feedback on our manuscript. |
| **Reviewer #1 Comments** | **Response** |
| 1. The sample size of this study is small, and the representativeness of the pregnant women's age, region, economic income, and occupation needs to be considered. It is advisable to include a description of the rationale behind the sample size selection, or to clarify the limitations of the small sample size in the discussion. | Thank you for your valuable comment and we appreciate the opportunity to clarify this feedback. In the revised manuscript we have now confirmed that the participants involved in this NGT study were recruited from our previous qualitative study under ***Study setting and selection of participants, see page 10 and Recruitment, see pages 11.*** This intentional continuity ensured that participants were already familiar with the context and contributed to developing the initial recommendations. Based on your comment we have now provided a clear explanation of the rationale for the sample size sample under ***Study setting and selection of participants, see page 10*** and explained how NGT methodology typically relies on small, purposive samples, prioritizing depth, interaction, and diversity of viewpoints rather than statistical generalizability. |
| 2. Using Ritchie framework analysis and NVivo coding, in line with qualitative research specifications, the consensus formation process needs to be supplemented with details, how to solve the parallel ranking. | Thank you for highlighting this and based on your valuable comment, we have added a matrix chart in ***S5 Tables*** to the already provided ranking tables for both groups ***(S3 Table and S4 Table).*** These matrix chart ***(S5 Tables)*** illustrates the ranking and refinement of recommendations across the two stakeholder groups. We also added additional information and explanation on the matrix chart under ***Data Analysis, see page 19*** detailing the multiphase analysis process. |
| 3. Refine data availability claims (e.g., repository name or access link) | Thank you for raising this important point. While participants provided verbal and written informed consent, including consent for using anonymized quotes in publications, ethical approval for this study did not extend to publishing the full interview transcripts per participant. This approach was explicitly discussed during pre-approval consultations with the Research Ethics Committees of the participating sites, where it was agreed that only selected, de-identified excerpts or participant quotes would be included in any dissemination materials to safeguard participant anonymity and confidentiality while minimizing the risk of deductive disclosure. This is particularly relevant in a healthcare setting involving professionals and service users from identifiable institutions. Therefore, consistent with established ethical guidelines and data protection principles, the manuscript presents only thematically relevant and anonymized quotes, using coded identifiers (e.g., M1 for postnatal mothers, LCC1 for healthcare professionals/Lactation Consultants) to preserve confidentiality while illustrating key findings.  The dataset contains sensitive, context-specific information drawn from a small sample, and even with pseudonymization, the risk of deductive disclosure remains. As a result, public access to full transcripts is restricted based on ethical considerations. The anonymized excerpts included in the manuscript are identified only by general codes (e.g., M1 for postnatal mothers, LCC1 for healthcare professionals) to preserve participant confidentiality. Requests for access to the underlying data may be submitted to the overseeing ethics body:  **Reviewing ethics committee**: Health Service Executive (HSE) Research Ethics Committees of three tertiary hospital sites in the Republic of Ireland (University Maternity Hospital Limerick- REC Ref: 119/2021, Rotunda Maternity Hospital Dublin-REC-2021-027, University Hospital Galway-Ref: C.A. 2718). Each of their contact details are in the approval letter for each site. |
| 4. Optimize language expressions, correct grammatical errors, and improve readability (e.g. whether "Addin' in a bit about the challenges" should be "Adding a bit about the challenges"). | Thank you for highlighting this. The manuscript has been closely proofread to ensure accuracy and clarity of language. We have thoroughly reviewed the manuscript to correct grammatical inconsistencies, refine phrasing, and ensure a consistent academic tone and readability. For example, ***see page first paragraph in page 26.*** |
| 5. If no clear ethical approval document is seen, whether the time of this study meets the ethical approval requirements. | Thank you for your comment regarding ethical approval. We would like to clarify that ethical approval for the NGT study was obtained through an approved original ethics applications submitted for our previous qualitative study and we have made this clearer under ***Ethical approval, page 19.*** We also made it clear under ***Recruitment (see page 11)*** that all participants were recruited from the ethical approved sites, and all study activities were conducted in full compliance with the requirements of the Research Ethics Committee (REC) and the Declaration of Helsinki.  We would like to thank you for your valuable feedback and we would like to express our sincere gratitude for your time and efforts in providing detailed and insightful comments on our manuscript. |
| **Reviewer #2 Comments** | **Response** |
| 1. Interview sample size, selection, and characteristics. The authors report that they selectively recruited participants who had participated in the prior qualitative study. What was the inclusion criteria in the previous study? How many participants were invited of which only 6 postnatal mothers agreed to participate? I’m concerned about the sample size, selection process, as well as the sample characteristics. The authors mention that the low breastfeeding rates underscore a pronounced cultural inclination towards formula feeding within Irish society. | - Thank you for your valuable comment and we appreciate the opportunity to clarify this. For this NGT phase, we purposefully re-invited participants from our previous qualitative study, in keeping with the study’s design, which aimed to refine and prioritise recommendations identified in our previous study, ensuring continuity and contextual relevance. We can confirm that the inclusion criteria for our previous qualitative study required that participants were (a) postnatal mothers who had attended prenatal breastfeeding education class within the selected research sites and (b) healthcare professionals directly involved in delivering prenatal breastfeeding education at the selected research sites (e.g., midwives or lactation consultants). This is the link to the qualitative study as cited in the revised manuscript: <https://doi.org/10.1371/journal.pone.0315269> - A subset of both postnatal mothers and healthcare professionals consented to participate in this NGT study **(*see Recruitment, page 11*).** The group sizes were consistent with established methodological guidance for NGT, supporting focused interaction and effective consensus-building within each session. We acknowledge that the sample size is modest but we have now explained the purposive nature of the recruitment and the rationale for methodological alignment with NGT under ***Study setting and selection of participants, see page 10.*** |
| Ethnicity may play a strong role in this case and 2 out of 6 postnatal mothers were Asian Indians. Also, in the text they mention Black Irish but in the table Irish and Black are separate categories. I’m wondering about the external validity of the study. | Thank you for commenting on ethnicity classification and its relevance to external validity. We acknowledge the importance of accurately and transparently representing ethnic identity in qualitative research. In this study, we followed the official ethnic classification system provided by the Central Statistics Office (CSO) of Ireland, which recognizes both "Black" and "Black Irish" as distinct and valid categories. The CSO allows individuals to self-identify within multiple options, including “White Irish,” “White Other,” “Black,” “Black Irish,” “Asian,” and others, to reflect the increasing diversity of the Irish population. The CSO acknowledges that not all individuals identifying as Black in Ireland would identify as Irish due to recent migration, citizenship status, or personal identification preferences. Therefore, our demographic table adopted this approach to ensure consistency with national data standards. |
| 2. Intro: “Irish-based studies on breastfeeding education have primarily focused on the relationship between breastfeeding education and outcomes such as initiation rates, duration, attitudes, and behaviours [16-19].”  What did these studies find? Briefly describe whether education had any significant (positive or negative) on these outcomes. | Thank you for noting this. We agree with your valuable comments and have now provided a brief summary of the key findings from the cited studies which are now cited as ***[18–20],*** highlighting the nature and significance of the relationship between breastfeeding education and relevant outcomes. This has been addressed under ***Introduction, pages 5 and 6***, which outlines whether the reported impact was positive, limited, or influenced by contextual factors. |
| 3. World Health Organization’s 10 Steps to Successful Breastfeeding is integral to the paper and I recommend presenting these steps in a table instead of supplementary file so that readers can relate to the discussion in the paper. | Thank you for this valuable comment. As per your suggestion we have created a new table showing the WHO’s 10 steps to Successful Breastfeeding, see ***Table 1, page 17.*** |
| 4. Methods section on scoring and ranking is confusing. Postnatal mothers assigned scores but did not rank the recommendations to my understanding. The scores ranged from 1-5 but number of options for each domain ranged from 2-4. There’s a considerable variation in how respondents scored i.e. in S3 table Question 2, participant 1 gave scores (2,1,4,4) whereas participant 2 gave scores (3,2,5,5). Authors need to give more details and clarify this difference in system of scoring. See scoring vs ranking on page 19 as well: “In the session with postnatal mothers, participants ranked the recommendations using a descending scoring system, assigning 5 points to their highest priority and 1 point to their lowest.” | Thank you for this valuable comment and based on your comment we have now clarified the scoring and ranking approach under ***Data analysis, page 18-19.*** We described how each postnatal mother assigned descending scores (5 to 1) to up to five recommendations per domain, resulting in variations depending on the available options. Aggregate scores were used to determine group priorities, with ties resolved by the frequency of votes. Healthcare professionals later assessed the feasibility of the prioritised recommendations using the same 5–1 scale. These clarifications are also reflected in ***S3 and S4 Tables*** which have now been revised these for clarity. |
| 5. The authors identified 7 recommendations as emerging from the initial list. Table S4 lists these. | Thank you for noting this and based on our comment we have now revised and updated the summary of findings Table S4 which is now ***S6 Table*** to ensure consistency and lists all 7 prioritised recommendations. |
| 6. Why does the summary in S5 have only 6 of these recommendations? | This discrepancy has now been corrected in the main manuscript under ***Key Recommendations and Consensus Outcomes from the NGT Sessions*** and ***Table 3 see page 22-24.*** |
| 7. The paper needs formatting? The section and sub-section headings are not clear. Please number them or clearly use a different text size or style to indicate the difference. The numbering of themes in the emergent themes section is inconsistent (1, 1.1, 2, 2.2, 3, 4, 5). Why not number them 1-7? | We thank and appreciate the reviewer for their attention to this matter. We have ensured that the paper is correctly formatted throughout. All sub-section headings and text size/style have been thoroughly revised, and all seven themes are numbered from 1 to 7 as you suggested under ***Emergent themes (in the Results section), pages 25-38.*** |
| Themes 1 and 1.1 were part of the same recommendation in the questionnaire as well as tables S4 and S5. Why were they split in the text? | Thank you for pointing this out and based on your comment we have now corrected this discrepancy under ***Emergent themes, pages 25-28.*** |
| The breakout room theme (part of question 3) is missing from this list altogether whereas this was identified as a priority by mothers. | Thank you for pointing this omission out and based on your comment we have now corrected this discrepancy and have included this theme under ***Emergent themes, pages 31-32.*** |
| 7. Section 2.1 is “Group Educational Sessions Featuring Real-Life Stories and Shared Experiences”but divergence and discordance discussion are around breakout rooms? | Thank you for pointing this out and based on your comment we have rectified this error under ***Emergent themes, pages 29-30.*** |
| 8. Section 3 on Ensuring Consistent and Accurate Breastfeeding Guidance (Pg 26) says “While mothers particularly valued standardized guidelines and a handbook to eliminate conflicting information, healthcare professionals emphasised training and mentorship equally. The healthcare professionals felt that hands-on training and ongoing mentorship would ensure that all staff members effectively interpret and apply the guidelines. “From table S3, handbook was ranked 3 out of 4 by mothers, why would you say that mothers particularly valued it when they didn’t? | Thank you for addressing this. Based on your comment, we have now corrected this discrepancy to accurately reflect the mother’s priorities as shown on the ranking table ***(See S3 Table).*** We have also revised the manuscript to reflect this under ***Emergent themes, pages 32-34.*** |
| 9. On page 27 “The divergence in emphasis reflects a difference in perspective on the mechanisms for achieving consistency—mothers prioritized a unified resource. At the same time, the healthcare professionals focused on a broader approach, including training and mentorship.” | Thank you for addressing this. Based on your comment, we have now corrected this discrepancy to accurately reflect that both groups agreed on the need for consistent and accurate guidance as shown on the ranking tables ***(S3 Table and S4 Table).*** We have also revised the manuscript to reflect this under ***Emergent themes, pages 32-34.*** |
| Mother did not prioritize a unified source as per table S3. Rather training was ranked second by them and unified source was ranked third after it. Mothers first priority was to establish standardized guidelines and clear consistent communication practices. | Thank you for addressing this. Based on your comment, we have now corrected this discrepancy to accurately reflect the mother’s priorities as shown on the ranking table ***(See S3 Table).*** We have also revised the manuscript to reflect this under ***Emergent themes, pages 32-34*.** |
| On page 30, addressing breastfeeding in public seems more discordant than divergence because the healthcare professions do not believe it fits into the current 10-step guidelines framework whereas the rest of the themes were fitting into the framework but just prioritized differently. | Thank you for this insightful observation. We agree that the interpretation of this theme as a discordance rather than a divergence is more accurate. Unlike the other themes aligned with the WHO’s Ten Steps to Successful Breastfeeding, the topic of breastfeeding in public revealed a fundamental misalignment. While postnatal mothers advocated for its structured inclusion in prenatal education, healthcare professionals questioned its fit within the WHO framework altogether. In response, we have revised the manuscript under ***Emergent themes, pages 36-38*** to reflect this as a clear discordance, highlighting the cultural specificity of the Irish context and the potential value of supplementary strategies to address this gap. |
| **Minor comments:**  1. Pg 5: “Participants included healthcare professionals (n=4) and postnatal mothers (n=4)” Postnatal mothers n=6? | Thank you for pointing this out and based on your comment we have now corrected this under ***Study setting and selection of participants, page 10 and Recruitment, page 11.*** |
| 2. Pg 6: “Moreover, community-level interventions promoting partner engagement and shifting societal perceptions were highlighted as essential components of a comprehensive strategy to improve breastfeeding outcomes”  Add period at the end of the sentence. | Thank you for pointing this out and based on your comment we have now corrected this under ***Introduction, page 8.*** |
| 3. Pg 11: “Data were collected on online on two separate days via MS Teams, approximately 3 weeks apart, with each online session lasting between 3.5 hours” Please check grammar | Thank you for pointing this out and based on your comment we have now corrected this under ***Data collection, page 12- 13.*** |
| 4. Pg 16: “Table 1 provides demographic details of all 6 postnatal mothers and 4 healthcare professionals (lactation consultants/ midwives) who participated in the study from three tertiary hospitals (n=10).  Please remove n=10 as it is misleading and redundant. | Thank you for pointing this out and based on your comment we have now corrected this under ***Results, page 20.*** |
| 5. Pg 16, Table 1: Age (mean) should be Age mean (SD)? | Thank you for pointing this out and based on your comment we have now corrected this under ***Results, see Table 2, page 20.*** |
| 6. Pg 18:  “The educational attainment of the healthcare professionals ranged from a Master’s degree (n=3;75%) and a Bachelor’s degree (n=1; 25%).”  “Most of the postnatal mothers were co-habiting (n 4; 66.67%), married (n=2; 33.33%) and identified as Irish (n=3; 50%), Black (Irish, African) (n=1; 16.67%) and Asian (Indian) (n=2; 33.33%).”  “Three of the postnatal mothers identified as in full-time employment (n=3; 50%), unemployed (n=2; 33.33%), and one mother (n=1; 16.67%) did not disclose her employment status.”  Please check grammar for all the sentences listed. | Thank you for pointing this out and based on your valuable comments we have now corrected these under ***Participant Characteristics (Healthcare Professionals and Postnatal Mothers), pages 21-22.*** |
| 7. Pg 19: “This approach entailed carefully triangulating the mothers’ consensus priorities with the healthcare professional’s feasibility evaluations, particularly noting alignment with the WHO’s ten steps to successful breastfeeding.”  Consider numeric 10 steps instead of ten steps as in the rest of the paper. | Thank you for pointing this out and based on your comment we have now revised the entire manuscript and corrected this using numeric 10 steps.  We would like to thank you for your valuable feedback and we would like to express our sincere gratitude for your time and efforts in providing detailed and insightful comments on our manuscript. |
| **Reviewer # 3** | **How and where it was addressed** |
| **Abstract**  If permissible by the journal, in the background of your abstract include the PMID when referencing your earlier study. | Thank you for this suggestion. According to the journal's guidelines, in-text references are not permitted in the abstract. Therefore, the earlier study has been appropriately cited in the main manuscript and included in the reference list. Here is a link to the study as cited in the manuscript:  <https://doi.org/10.1371/journal.pone.0315269> |
| **Introduction**  Does 40% at three months mean exclusively breastfed or does it include combo feeding? make that clear throughout if you mean exclusive (as in no introduction to formula) | Thank you for your valuable comment. We have now clarified this in the manuscript by stating that 40% breastfeeding rate at three months includes any form of breastfeeding (both exclusive and partial breastfeeding) under ***Introduction, page 4.*** |
| - what is the highest breastfeeding initiation rate in Europe for comparison? | Thank you for pointing this out and based on your comment we have now corrected this under ***Introduction pages 4-5,*** comparative breastfeeding initiation rates from selected European countries are included in the manuscript to provide context for Ireland’s standing. |
| - Checked your numbers from the 2020 report and the stat for 2020 actually says 62.3% not 60%. Please double check your numbers for the other statistics as I don't have access to the journal for the other statistics | Thank you for pointing this out and based on your comment we have now corrected this under ***Introduction page 5***. Rather than using the 2020 Irish Maternity Indicator System Statistics, we have updated the manuscript to reflect the most recent data from the 2023 Irish Maternity Indicator System (IMIS). See ***(Introduction page 5).*** |
| Your intro mentions a need for personalization with breastfeeding education, are these programs you're referring to talking about one-on-one lactation consultant education, prenatal education from your hospital, education immediately postpartum, group education? Please specify. | Thank you for your question. The statement in the introduction refers specifically to personalisation within group-based prenatal breastfeeding education classes, as identified in our previous qualitative study. Mothers desired tools like pre-class surveys to tailor content to their concerns and experiences, creating personalized learning pathways within a group setting. This clarification has now been incorporated into the revised manuscript for greater specificity ***(see Introduction, page 7).*** |
| **Recruitment**  Although rapport and familiarity are nice, I don't know given the nature of your study necessitated it. | Thank you for your valuable comment. We agree with you that rapport should not compromise research rigour and have carefully considered its role in our study design. In this case, participants’ prior involvement in the earlier qualitative phase did not bias but enhanced the depth and authenticity of discussion in the NGT sessions. The literature supports the value of established rapport in qualitative research, particularly in iterative, multi-phase designs, where familiarity can facilitate openness, trust, and richer participant contributions (Knox & Burkard, 2009; Guillemin & Gillam, 2004). This is especially relevant when exploring emotionally sensitive issues such as infant feeding, where trust can support more candid and reflective engagement. Therefore, we have retained the statement in the manuscript under ***Introduction, page 11.***  Guillemin, M., & Gillam, L. (2004). Ethics, reflexivity, and “ethically important moments” in research. Qualitative Inquiry, 10(2), 261–280. <https://doi.org/10.1177/1077800403262360>  Knox, S., & Burkard, A. W. (2009). Qualitative research interviews. Psychotherapy Research, 19(4–5), 566–575. <https://doi.org/10.1080/10503300802702105> |
| How did you determine that 10 participants were enough to collect this type of information? Did you feel like you reached saturation with your data collection? | - Thank you for your valuable comment and we appreciate the opportunity to address this. We have now revised the rationale for the sample size in the manuscript and confirmed that the participants involved in this NGT study were recruited from our previous qualitative study under ***Study setting and selection of participants, page 10***. This intentional continuity ensured that participants were already familiar with the context and contributed to developing the initial recommendations. Based on your comment we have now provided a clear explanation of the rationale for the sample size sample under ***Study setting and selection of participants, see page 10*** and explained how NGT methodology typically relies on small, purposive samples, prioritizing depth, interaction, and diversity of viewpoints rather than statistical generalizability. - Regarding data saturation, it is important to note that saturation is not a required outcome in structured consensus methods such as NGT, where the primary aim is not thematic exhaustiveness, but rather the generation, ranking, and feasibility testing of stakeholder-informed priorities (Harvey & Holmes, 2012). Therefore, the structured design of the sessions ensured that each participant contributed fully to the refinement and evaluation process. We hope this explanation and the updated manuscript text address your concern regarding sample adequacy and methodological rigour. - Harvey, N., & Holmes, C. A. (2012). Nominal group technique: An effective method for obtaining group consensus. International Journal of Nursing Practice, 18(2), 188–194. <https://doi.org/10.1111/j.1440-172X.2012.02017.x> |
| Also, curious the breastfeeding histories of the moms that participated. I imagine there would be differences in opinion for the woman who did extended breastfeeding vs. the woman who didn't continue after leaving hospital. | Thank you for your valuable comment. While we acknowledge that individual breastfeeding histories may influence perspectives, the objective of this NGT study was not to examine personal trajectories but to refine and prioritize recommendations developed in our previous qualitative study (Kehinde et al., 2024). Our previous study focused on the perspectives of healthcare professionals and postnatal mothers regarding prenatal breastfeeding education classes in Ireland and their influence on mothers’ decisions to breastfeed. The findings shaped the development of the recommendations brought forward in this NGT study. In this NGT study, the emphasis was on achieving structured group consensus around priority recommendations rather than further exploring individual feeding histories.  See link to the previous study as cited in the revised manuscript: <https://doi.org/10.1371/journal.pone.0315269> |
| **Data collection**  Between what and 3.5? was the second session shorter or longer 3.5? | Thank you for noting this and we fully agree with you. Based on your comment we have now revised this ***under Data collection, page 12-13,*** and clarified that the two sessions were conducted separately — one with postnatal mothers and the other with healthcare professionals — and lasted approximately three and a half hours. |
| If participants were going to silently rank and review, why choose a group format and not just a survey with follow up individual interviews (if needed)? What benefit was there to using a focus group vs another method for this type of data collection? | Thank you for your valuable comment and we appreciate the opportunity to clarify this. The NGT was intentionally selected over surveys or individual interviews because it offers individual reflection and structured group interaction, central to its strength as a consensus-building method. Although participants initially ranked recommendations independently, the group format allowed for discussion, clarification, and justification of preferences, which can reveal underlying reasoning and collective priorities that might not emerge in isolated responses. Unlike surveys, NGT provides a collaborative yet time-bound setting that enables stakeholders to express and negotiate the relative importance of ideas through dialogue. This setting is ideal for refining and prioritising stakeholder-informed recommendations developed in our previous qualitative study. Furthermore, NGT reduces the dominance of any single voice by giving all participants equal opportunity to contribute during idea generation and voting and avoids the interpretive bias often present in unstructured focus groups. This structured format is particularly well-suited to health services research, where practical, actionable consensus is a key outcome. We have given clear rationale for using NGT under ***Introduction, page 8, Methods, page 9-10*** |
| **Data Analysis**  Did you use verbatim transcription for NVIVO? | Thank you for your question. Yes, the NGT sessions were audio-recorded and transcribed verbatim prior to being imported into NVivo (Version 14; QSR, 2024) for data management and coding. Based on your valuable comment we hance now clarified this point under ***Data analysis page 18*** to ensure transparency in our data preparation process. |
| Table 1: In the United States (my location), Black is considered a "race" not an "ethnicity". Does "Irish" mean white here? White would be considered a race. Did your Black participant not come from Ireland? What does any other "white" background mean, like from another European country? Consider a restructure of this variable and break into two variables with 1 for ethnicity if any participants were citizens from another country than Ireland and the other variable to be Race (White, Black, Asian, Another race). Not necessary to note your Asian participant was Indian unless she's from India. - Marital status- nobody was living solo? - I can see the benefit of having all the demographics in one table, however I would consider reorganizing the variable order by adding row headers for the mothers and the staff and placing variables under their respective headers. | - Thank you for your valuable comments on this and we appreciate your perspective and acknowledge that definitions of race and ethnicity vary internationally. In this study, we followed the official ethnic classification system provided by the Central Statistics Office (CSO) of Ireland, which distinguishes between categories such as "White Irish," "White – Other Background," "Black," "Black Irish," and "Asian". This system reflects self-identified ethnicity rather than race and is widely used in Irish health and social research to ensure consistency with national data reporting standards. As per CSO guidance, "Black" and "Black Irish" are considered distinct categories to reflect diversity in migration, citizenship, and personal identity. For example, not all individuals identifying as Black in Ireland also identify as Irish—some may be recent migrants or may not hold Irish citizenship. For this reason, we maintained the original categorisation to respect participant self-identification and align with Irish demographic norms. - Regarding the structure of Table 1, which is now ***Table 2 (page 20-21),*** this revised table includes separated headers for postnatal mothers and healthcare professionals, with variables placed accordingly. We have reviewed this layout to ensure it is distinguishable. - On marital status, all participants in this study were either married or cohabiting. No participants identified as living alone. To avoid ambiguity, we have clarified this point in the table footnote ***(see Table 2, page 21).*** - Lastly, we acknowledge the suggestion to reformat by separating race and citizenship; however, given that citizenship data were not collected, and that ethnicity was captured following national standards, we have retained the existing format to preserve data accuracy, participant self-identification, and alignment with official Irish classifications. |
| Ideas generated:  -read again to make sure you're not repeating your methods section, omit any repetition | Thank you for your valuable suggestion and as you suggested we have carefully reviewed this section which is now titled ***“Key Recommendations and Consensus Outcomes from the NGT Sessions”*** and removed any repetition under ***Results, pages 22-24*** to improve clarity and avoid redundancy. |
| **Results:**  - I think there is value in including just a very high-level summary table of the WHO 10 steps (just what each step is would be sufficient) for reference so someone doesn't have to google it to understand what you are specifically referencing throughout. | Thank you for your valuable suggestion and we fully agree that this would enhance clarity for the reader. Therefore, we created a new table of the WHO Ten Steps to Successful Breastfeeding see ***Table 1, page 17.*** |
| It sounds like your healthcare providers are not particularly enthusiastic around public breastfeeding and would only discuss if they needed to base on the quotation selected. Does that feel like an accurate interpretation? | Thank you for pointing this out we appreciate the opportunity to clarify this. The selected quotation may have unintentionally given the impression that healthcare professionals were reluctant to engage with the topic of public breastfeeding. However, our findings indicate that healthcare professionals acknowledged the importance of this issue and its cultural sensitivity and supported its inclusion in prenatal education. However, they questioned its alignment within the formal structure of the WHO Ten Steps to Successful Breastfeeding. Therefore, their reservations were not based on a lack of enthusiasm but rather on structural and curricular constraints. This led them to suggest a more informal, discussion-based integration rather than a formal component. Based on our valuable comment we have clarified this distinction ***under Emergent themes, page 36-38.*** |
| Discussion:  Breastfeeding Peer learning isn't mentioned until the recommendations section but is an evidence-based approach to breastfeeding education. Consider expanding on that a little more in the discussion when talking about the importance of storytelling. | Thank you for your valuable comment and we appreciate the opportunity to clarify this. We agree that peer learning is an important, evidence-based approach to breastfeeding education and complements the emphasis on storytelling in our findings. Therefore, we have expanded on this under the ***Discussion, page 40-41*** which briefly highlights the relevance of storytelling and shared experiences, supported by existing literature, as a valuable component of the prenatal breastfeeding education model. |
| Fig 1:  You used a step-wise program, but this branching figure doesn't apply time particularly well, given that the arrows go east/west rather than down. Please restructure for clarity. | Thank you for your valuable suggestion and we appreciate the opportunity to clarify this. This Figure 1 is intentionally structured to illustrate the parallel processes of postnatal mothers and healthcare professionals during the NGT sessions. While the layout is horizontal for visual clarity, the sequence of steps remains linear, time-bound, and delineated. The central convergence points highlight integrating both groups’ contributions into a unified, consensus-based set of recommendations. We believe the figure accurately and effectively communicates the process and have retained it in its current form. |
| Fig 2 Does not seem as necessary to me considering it is clearly outlined within the paper itself. | Thank you for your valuable comment and as you suggested we have removed the referenced figure, as its content is clearly outlined in the main text. This adjustment was made to ensure clarity and reduce redundancy within the manuscript.  We would like to thank you for your valuable feedback and we would like to express our sincere gratitude for your time and efforts in providing detailed and insightful comments on our manuscript. |
