# Supplementary material for: Validating and prioritizing prenatal breastfeeding education recommendations: A nominal group technique study with postnatal mothers and healthcare professionals
Source: PLoS One. 2025 Jul 16;20(7):e0328542. doi: 10.1371/journal.pone.0328542 (PMC12266410; doi:10.1371/journal.pone.0328542)
Supplement: Attachment — Submitted filename: Response to Reviewer 1.docx [file pone.0328542.s012.docx]

**Manuscript Ref. No: PONE-D-25-09271R1**

**Responses to reviewers’ comments on Title:** *Validating and Prioritizing Prenatal Breastfeeding Education Recommendations: A Nominal Group Technique Study with Postnatal Mothers and Healthcare Professionals.*

We would like to express our sincere gratitude to the editor and the reviewers for their time and efforts in providing detailed, insightful, and valuable feedback on our manuscript. Their suggestions have helped to sharpen our thinking and, we hope, led to a substantially improved submission based on their valuable constructive feedback.

| **Editor’s Comments** | **Response** |
| --- | --- |
| **General Journal Requirements**  Please review your reference list to ensure that it is complete and correct. If you have cited papers that have been retracted, please include the rationale for doing so in the manuscript text, or remove these references and replace them with relevant current references. Any changes to the reference list should be mentioned in the rebuttal letter that accompanies your revised manuscript. If you need to cite a retracted article, indicate the article’s retracted status in the References list and also include a citation and full reference for the retraction notice. | We have carefully reviewed the reference list and confirm that all citations are complete, accurate, and up to date. No retracted articles are cited in the manuscript, and no changes have been made to the reference list. |
| **Reviewer #1 Comments** | **Response** |
| 1.Sample Representativeness: While the use of a purposive sample is justified for qualitative NGT studies, the authors should further acknowledge the limited generalizability of the findings and discuss this as a study limitation. | Thank you for your insightful comment and we appreciate your observation. Based on your valuable comment, we have strengthened and clarified this point by explicitly linking the use of a purposive sample to limited generalisability and framing this as a methodological limitation. Please see revised text under **Limitations, page 52** |
| 2. Language and Structure: The manuscript is written in generally clear English, but some segments—especially those with extensive participant quotations—could benefit from slight condensation or thematic grouping for better clarity and flow. | Thank you for highlighting this and we appreciate the opportunity to clarify your valuable comment. The quotations are currently grouped under clearly defined themes and subheadings, with each quote selected to represent unique or consensus views. All quotations were presented verbatim, in keeping with qualitative standards, and were thematically integrated with interpretive narrative. In response to your valuable comment, we conducted a careful review of the Results section. Where appropriate, we condensed repetitive or overlapping segments by summarising similar quotes in narrative form, and we applied minor formatting adjustments (e.g., punctuation) to enhance clarity without altering the meaning or integrity of participant voices. Please see these changes under **Results, pages 25-37.** |
| 3. Figures and Tables (Optional):Consider including a visual summary (e.g., a flowchart of the NGT process or a radar chart summarizing consensus priorities) to enhance readability and support international readers’ comprehension. | Thank you for highlighting this and we appreciate the opportunity to clarify your valuable comment. In accordance with PLOS ONE guidelines, we have included the recommended visuals, specifically, a figure outlining the NGT process and a table summarising stakeholder consensus priority under the **Supporting Information section.** These materials are clearly referenced in the manuscript, please see ***Fig 1: NGT process*** (page 13); and ***S7 Table*** (page 25).  Many thanks for you feedback and we would like to express our sincere gratitude for your time and efforts in providing detailed, insightful, and valuable feedback on our manuscript. |
